# Unexpected increase in the oxidation capacity of the urban atmosphere of Madrid, Spain

**DOI:** 10.1038/srep45956

**Published:** 2017-04-11

**Authors:** A. Saiz-Lopez, R. Borge, A. Notario, J. A. Adame, D. de la Paz, X. Querol, B. Artíñano, F. J. Gómez-Moreno, C. A. Cuevas

**Affiliations:** 1 Department of Atmospheric Chemistry and Climate, Institute of Physical Chemistry Rocasolano, CSIC, Madrid, Spain; 2 Laboratory of Environmental Modelling, Department of Chemical and Environmental Engineering, Escuela Técnica Superior de Ingenieros Industriales, Technical University of Madrid (UPM), Madrid, Spain; 3 University of Castilla-La Mancha, Physical Chemistry Department, Faculty of Chemical Science and Technologies, Ciudad Real, Spain; 4 Atmospheric Sounding Station-El Arenosillo, National Institute for Aerospace Technology (INTA), Atmospheric Research and Instrumentation Branch, Mazagón-Huelva, Spain; 5 Institute of Environmental Assessment and Water Research, CSIC, Barcelona, Spain; 6 Environmental Department of the Research Center for Energy, Environment and Technology (CIEMAT), Madrid, Spain

## Abstract

Atmospheric oxidants such as ozone (O_3_), hydroxyl and nitrate radicals (OH and NO_3_) determine the ability of the urban atmosphere to process organic and inorganic pollutants, which have an impact on air quality, environmental health and climate. Madrid city has experienced an increase of 30–40% in ambient air O_3_ levels, along with a decrease of 20–40% in NO_2_, from 2007 to 2014. Using air pollution observations and a high-resolution air quality model, we find a large concentration increase of up to 70% and 90% in OH and NO_3_, respectively, in downtown Madrid (domain-wide average increase of 10% and 32% for OH and NO_3_, respectively). The results also show an 11% reduction in the nitric acid concentrations, leading to a remarkable denoxification of this urban atmosphere with implications for lower PM_2.5_ levels and nitrogen input into ecosystems. This study suggests that projected worldwide NO_x_ emission reductions, following air quality standards, will lead to important changes in the oxidizing capacity of the atmosphere in and around large cities.

Nitrogen oxides (NO_x_ = NO + NO_2_) ambient concentrations can have important implications for human health^1^ and have become a relevant target of environmental policies for many cities worldwide^2^,^3^,^4^. NO_x_ emissions are directly related to combustion processes, including mobile sources that are particularly relevant for urban environments, where exceedances of NO_2_ human protection thresholds occur frequently^5^. Many cities in Europe are having difficulties to meet the NO_2_ standards established by the European Air Quality Directive^6^. The European Environment Agency (EEA)^5^ reported that 8% of the EU population are exposed to NO_2_ levels that exceed European and World Health Organization (WHO) air quality targets. Recent observations have reported reductions of NO_2_ concentrations in urban areas following the application of environmental policies and the effects of recent economic recessions^2^,^4^,^7^. In the EU, it was reported that the 2003–2012 decrease was −18% (−0.4 and −0.7 μg NO_2_/m^3^/year for urban and traffic sites (EEA 2014), which was markedly lower than that of the EU NO_x_ emission inventory for the same period (−30%), due to the preferential decrease of NO versus NO_2_. Combined ground- and satellite-based measurements carried out from 1996 to 2012 have shown a dramatic decrease in tropospheric NO_2_ over the largest and most industrialized cities of Spain, including Madrid^8^. Remarkably, this decrease was more abrupt from 2008 to 2012 attributed primarily to emissions cut downs due to the economic recession^8^. Nevertheless, the efficient role of local emission abatement measures in the recently observed reduction of NO_2_ ambient levels in Madrid has also been suggested^9^.

NO_x_ levels influence O_3_^10^ and other photochemical pollutants^8^,^11^, for example as precursors of the NO_3_ radical, the main oxidant of the nocturnal atmosphere^12^,^13^,^14^,^15^. Hence, changes in NO_x_ emissions may have important impacts on the chemistry of an urban atmosphere and its oxidizing capacity, i.e. the efficiency of the atmosphere to oxidize and ultimately remove the large variety of organic and inorganic species that are emitted into it from both anthropogenic and natural sources. Over recent years, observations show a general increasing trend of O_3_ concentrations in European urban areas^5^. This has been mostly attributed to: *i*) the occurrence of these trends in volatile organic compounds (VOCs)-sensitive areas (where NO_x_ reduction might increase O_3_ levels) (EEA, 2014); or *ii*) the decrease of the NO/NO_2_ emission and ambient air ratios, what in turn reduces the O_3_ consumption (titration effect) by NO^16^.

Besides being a greenhouse gas and have harmful implications for vegetation and human health^17^,^18^, tropospheric O_3_ is also the main source of OH radicals, the most influential tropospheric oxidant in daytime atmospheric chemistry. The OH radical initiates many oxidation reactions in the troposphere, leading to the formation of new secondary photochemical smog pollutants, including more O_3_. The oxidation of organic compounds by OH can also enhance the formation of secondary organic aerosols (SOA), which has implications for environmental health and climate^19^,^20^.

During the night, the concentration of OH is drastically reduced and the oxidizing capacity of the atmosphere is then controlled by NO_3_ (together with ozone, which is also an important tropospheric oxidant). The amount of VOCs oxidised by NO_3_ at night is comparable to that due to OH during the day^21^. The NO_3_ radical is also a very important chemical sink for isoprene and other biogenic VOCs, whose atmospheric oxidation results in the formation of SOA precursors^5^.

Here we combine observations and a state-of-the-art mesoscale air quality modelling system (AQM) to assess the impact of the observed rapid change in NO_x_ emissions and O_3_ levels, recorded during 2007–2014, on the chemistry and oxidizing capacity of the urban atmosphere of Madrid. We assume that 2007 is representative (as baseline) for recent changes in emissions and air quality, because it corresponds to the last year before the economic recession, and the implementation of Madrid’s air quality plan intended to meet NO_2_ ambient concentration standards. Also 2007 does not present exceptional meteorological features.

## Results

### NO_x_ concentration trends

**Figure 1:**
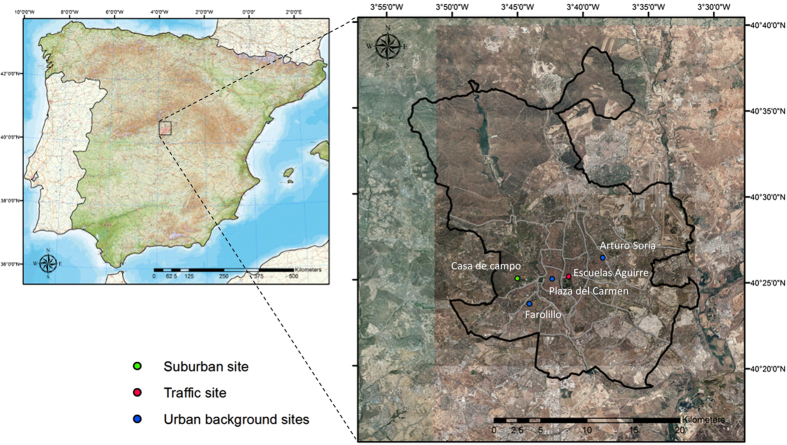
Localization within the Iberian Peninsula and general view of the modelling domain including the location of the air quality monitoring stations selected for this study. The black line represents the limits of the Madrid municipality. Figure created by the authors using ArcGis 10 (http://www.esri.com/software/arcgis/arcgis-for-desktop) and maps from ©Instituto Geográfico Nacional of Spain (http://centrodedescargas.cnig.es/CentroDescargas/).

Five representative monitoring stations from the Air Quality Network (AQN) of Madrid were selected to carry out this study (Fig. 1). The selected stations are adequate to investigate the air quality evolution in the Madrid metropolitan area since they are typical of locations directly influenced by intense traffic emissions (Escuelas Aguirre), urban background (Plaza del Carmen, Arturo Soria and Farolillo) and suburban (Casa de Campo) environments. This allows assessing the influence of different emission sources on pollutant concentrations under a variety of temporal and spatial atmospheric phenomena. Note that unlike the rest of monitoring stations of the Madrid AQN, they have not been relocated and have a complete record of hourly NO_x_ and O_3_ data for the period of interest (>98% hourly data coverage), thus providing a consistent view of air quality evolution in the city.

**Figure 2:**
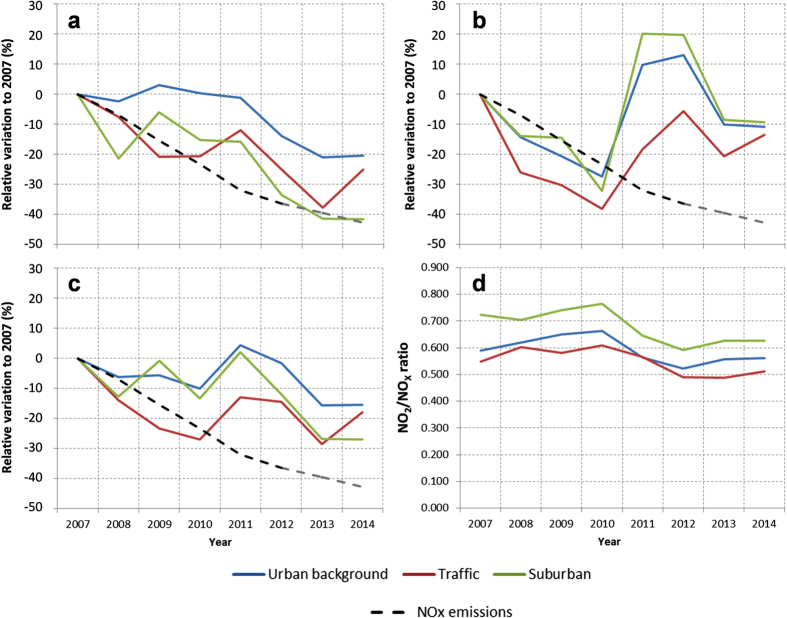
Ambient air concentration change (relative to levels of 2007) of (**a**) NO_2_; (**b**) NO and (**c**) NO_x_. Dashed line corresponds to the variation of NO_x_ emissions in Madrid during the same period. (**d**) NO_2_/NO_x_ ratio measured from 2007–2014.

A decrease of 20–40% in NO_2_ levels was registered by the different stations during 2007–2014 (3–6%/year Fig. 2a), with a mean change rate of −1.6 and −2.7 μgNO_2_/m^3^/year for urban background and the traffic sites, respectively. These decreasing rates are much more pronounced than the EU averaged rates for the same period and type of urban sites reported by the European Environmental Agency^22^: −0.4 and −0.7 μgNO_2_/m_3_/year, respectively (2%/year). Other studies^8^,^23^ reported a reduction of 37% in the 1996–2012 period (2.3%/year). In the case of NO, the measurements show a decrease around 10–15% in 2014 respect to the measured levels in 2007 (Fig. 2b). A decrease in the NO_2_/NO_X_ ratio is observed in Madrid during the period of analysis although positive slopes occur on individual years. This effect cannot be explained only by NO_x_ emission trends (represented by dashed lines in Fig. 2), since they follow a clear monotonic decreasing tend over the period of interest. Further inspection of emission datasets reveal that no substantial changes on the speciation of NO_X_ emission exist during the period analysed. Hence, the NO increases observed for 2011 and 2012 may be influenced by variations in local meteorological conditions that led to particularly high NO levels in winter (Fig. S1). Analysis of the monthly temperature during the cold months (November to February) of 2011 and 2012 reveals similar values to those of the rest of the data series, between 5 and 10 °C. Monthly mixing layer height does not show significant differences, with values between ~650 m in December and ~1000 m in February, similar to the other years. However, the wind speed data shows stagnant periods of calm winds during the winters of 2011 and 2012 (Fig. S2). Additionally, two-temperature measurements of deep surface inversions confirm these strong stability conditions (Fig. S2), which are not revealed by estimations of mixing layer height provided by models. These conditions favour primary NO accumulation while low temperature and radiation limit its oxidation to NO_2_, consistent with the NO_2_/NO_X_ decrease observed in Fig. 2d. Therefore, meteorological factors such as major atmospheric stagnation and resulting less atmospheric dispersion in the lower layers could influence the anomalous NO_2_/NO_X_ behaviour recorded during these two years. Although these phenomena may deserve a more specific analysis, this study focuses on the net internal variation of air quality over the city, which minimizes the influence of local factors and provides more robust and policy-relevant information.

NO_x_ levels are 15–30% lower in 2014 than in 2007 (Fig. 2). Total NO_x_ emissions according to the Madrid’s local emission inventory^24^ have dropped from 20839 t yr^−1^ in 2007 to 13264 t yr^−1^ in 2012, last year available. According to emission projections, two years later the emissions have further dropped to 11913 t yr^−1^ in 2014^9^. This represents a 42% decrease in NO_x_ emissions between 2007 and 2014. These trends are strongly related to emission reductions in the road traffic sector, that globally contributes to 59% of ambient NO_2_ concentration levels in the modelling domain used in this study (up to 90% in the city centre). Anthropogenic VOCs emissions (93% of total VOCs in 2007) are reduced by 7% in that period in the modelling domain. Further details on emissions scenario computation and source apportionment can be found elsewhere^9^. The observed nonlinearities between NO_x_ emissions and NO_2_ trends in Madrid have also been the subject of evaluation in previous studies^8^,^25^. Dispersion processes and NO_x_ speciation, as well as other local factors such as the influence of background levels, affect differently to the diverse emission sources throughout the city so that the effect of emission reductions is not proportional to the observed air quality levels.

**Figure 3:**
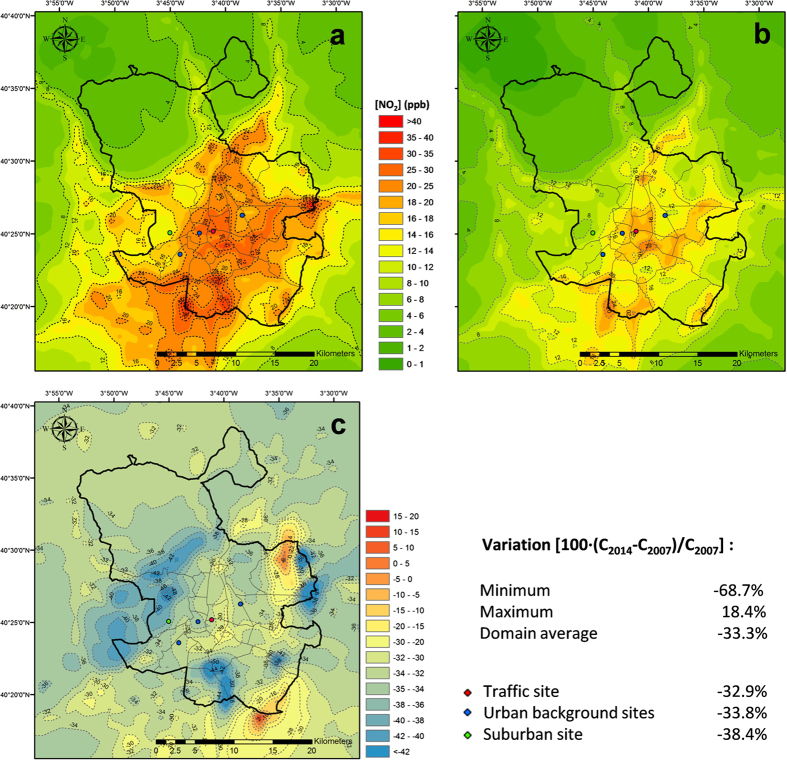
Modelled NO_2_ annual mean concentration, (**a**) 2007; (**b**) 2014. (**c**) Variation of NO_2_ annual mean concentration in 2014 with respect to 2007 and resulting statistics. Positive values indicate concentration increase. Figure created by the authors using ArcGis 10 (http://www.esri.com/software/arcgis/arcgis-for-desktop).

The CMAQ (Community Multiscale Air Quality) mesoscale chemical-transport model^26^,^27^,^28^,^29^ is used here to simulate pollutant trends and its associated atmospheric impacts not only at the location of air quality monitoring stations but over a modelling domain that covers the entire metropolitan area. Analysis of the CMAQ results (Fig. 3) shows a NO_2_ decrease of 32–38% across the different typology stations, and 33% over the entire metropolitan area, in good agreement with the decrease (20–40%) measured by the AQN in Madrid (Fig. 1).

### O_3_ concentration trends

**Figure 4:**
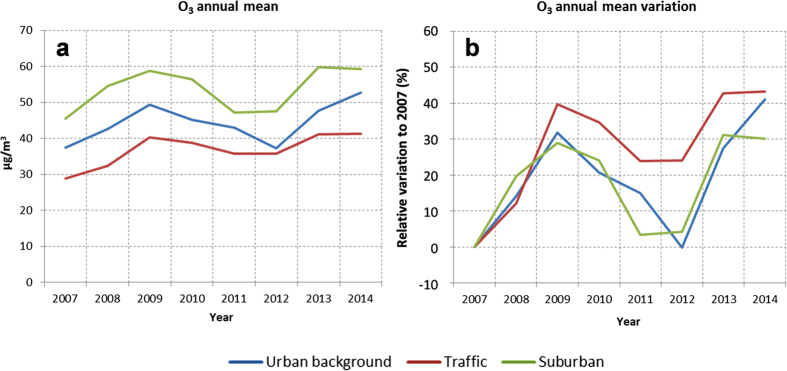
(**a**) Average ambient concentration levels of O_3_ recorded at representative monitoring stations of Madrid during the period 2007–2014. (**b**) Percentage variation of ozone concentrations respect to levels of 2007.

**Figure 5:**
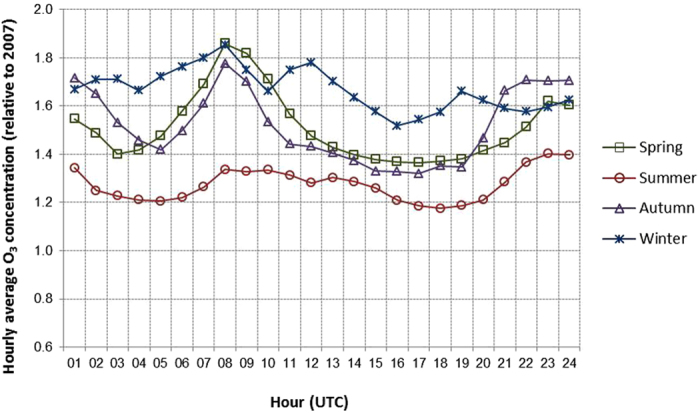
Evolution of the daily average concentration profile of O_3_ from 2007 to 2014 (relative to 2007 with a value = 1) in the urban background monitoring stations used in the present work.

Figure 4 shows the remarkable increase in annual averaged O_3_ levels during this period. Increases of more than 10 μg m^−3^ as integrated annual levels are measured in 2014 by comparison with 2007 throughout all monitoring stations (Fig. 4a). The slope of the corresponding trends during 2007–2014 demonstrates a general positive progression of O_3_ concentrations over Madrid. The percentage of O_3_ concentration change in 2014 with respect to the 2007 levels ranges from 30% to 43% in suburban and traffic stations, respectively, (Fig. 4b). The evolution of the daily average concentration profile of O_3_ in the period 2007–2014 shows an increase throughout the day for all seasons, as represented in Fig. 5, where evolution of the daily average levels of ozone from 2007 to 2014 (data relative to 2007) is plotted. The relative 24 h-integrated increment is particularly noticeable for winter (66% as an average). Maximum O_3_ differences of up to 80% occur around the morning traffic rush hours (up to 30 μg m^−3^ in spring). This is due to the strong reduction in NO emissions from road traffic and the resulting decrease in O_3_ titration efficiency. The lack of such relative maximum in summer, when road traffic intensity declines, supports this hypothesis. A second increase in O_3_ levels is also evident during the evening, which would be attributed to the reduction in NO_x_ levels, typically high at this time of day in Madrid (Fig. S3).

**Figure 6:**
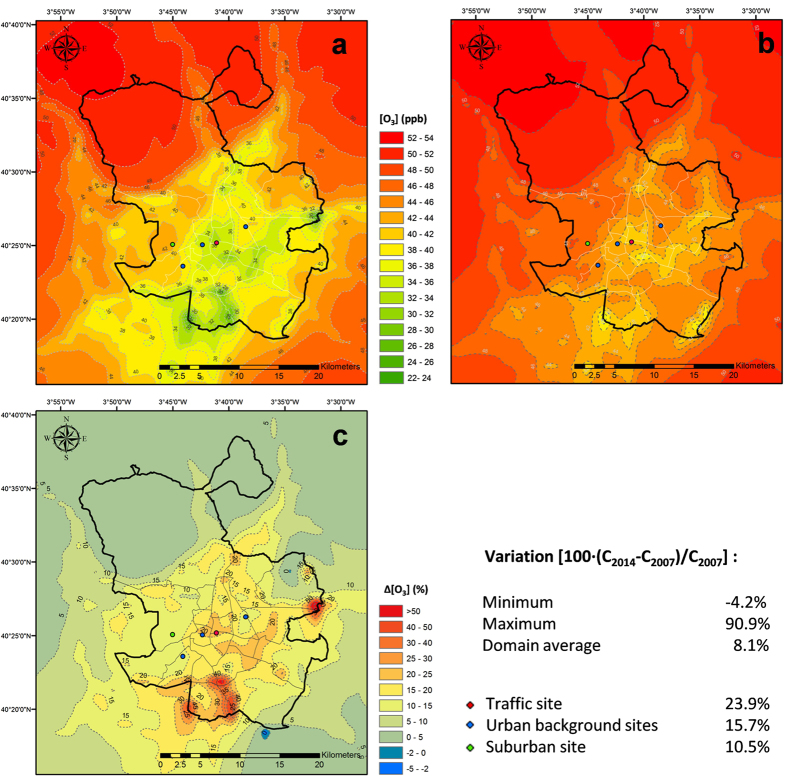
Modelled O_3_ annual mean concentration, (**a**) 2007; (**b**) 2014. (**c**) Variation of O_3_ annual mean concentration in 2014 with respect to 2007 and resulting statistics. Figure created by the authors using ArcGis 10 (http://www.esri.com/software/arcgis/arcgis-for-desktop).

Output of the CMAQ model at the site of the AQN stations shows an increase of O_3_ concentrations that ranges from 10 to 24% depending on the location of the stations (Fig. 6). For the specific location of measurement sites, the modelled O_3_ percentage increase is lower than that observed (30–40%). However, Fig. 6c shows that when integrated over the modelling domain, the simulated average O_3_ variation is 8%, with a mean value of 30% over the downtown area, in better agreement with the measured percentage change (Fig. 4).

### Impact on atmospheric oxidants

**Figure 7:**
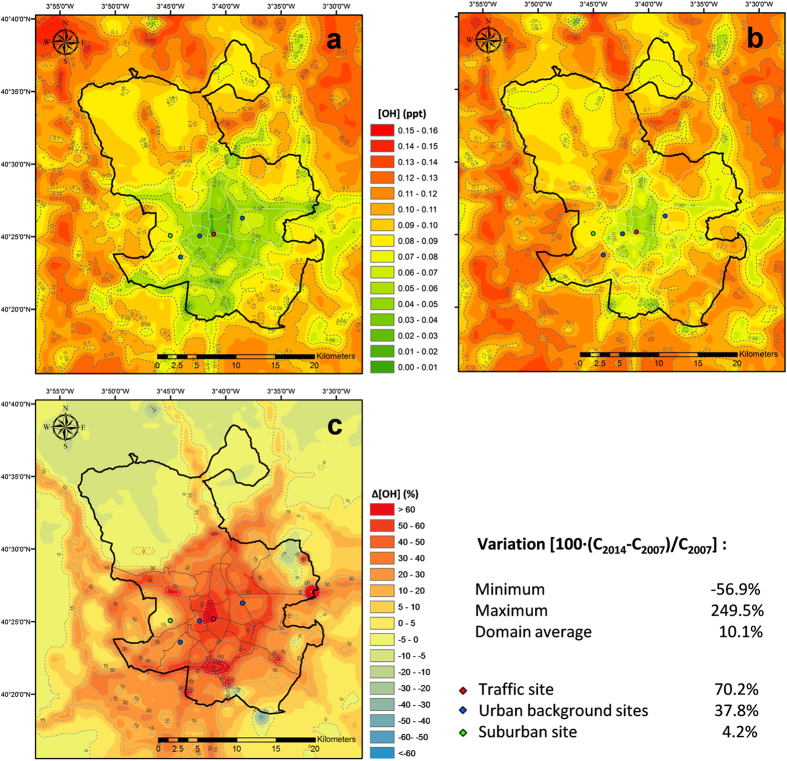
Modelled OH annual mean concentration, (**a**) 2007; (**b**) 2014. (**c**) Variation of OH levels in 2014 with respect to 2007. Figure created by the authors using ArcGis 10 (http://www.esri.com/software/arcgis/arcgis-for-desktop).

We now turn to the influence of the observed O_3_ increase, combined with the reduction in NO_2_ levels, upon the concentrations of the main atmospheric oxidants (OH and NO_3_). Our results show that the sharp increase observed in O_3_ levels during the 2007–2014 period leads to a significant concentration rise in OH, which forms by photolysis of O_3_ and subsequent reaction with water vapour. However, additional factors may have also affected OH. For example, if NO_x_ levels decrease, the importance of the NO_2_ + OH reaction also decreases. This, in turn, can increase OH since the reaction consumes less OH. Annual mean OH levels increase about 50% in the downtown area, between 4% and 70% across the different typology of the monitoring stations, and an average of 10% over the entire modelling domain (Fig. 7). Note that the modelled variation in OH is likely a lower limit since the model slightly under predicts the observed O_3_ trend (Fig. 6). Regarding the daily behavior of OH and NO_3_ radicals, daily curves are very similar for 2007 and 2014, although differences are observed as a function of stations typology (Figure S4 at Supplementary).

**Figure 8:**
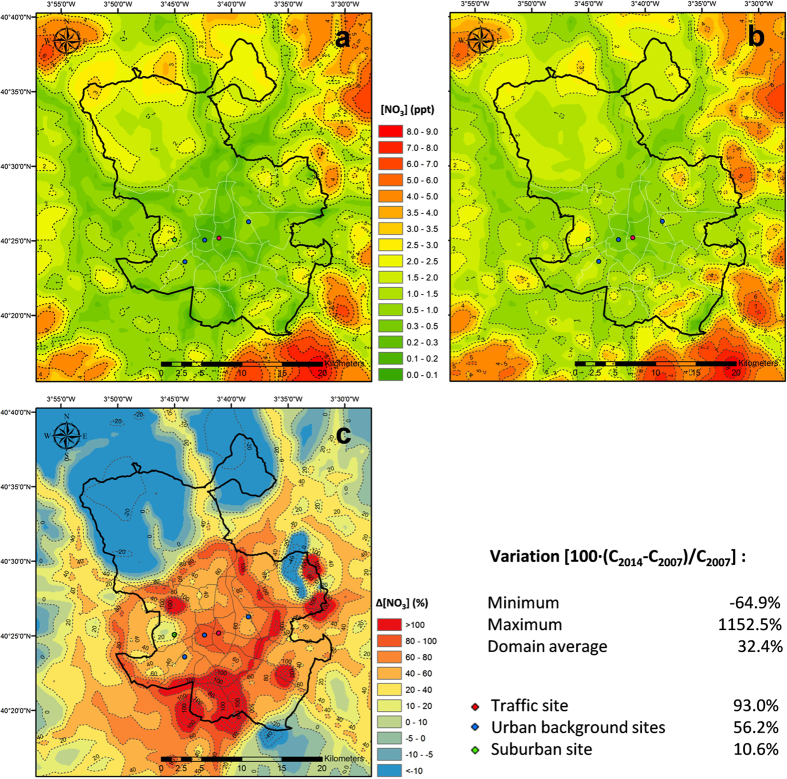
Modelled NO_3_ annual mean concentration, (**a**) 2007; (**b**) 2014. (**c**) Variation of NO_3_ levels in 2014 with respect to 2007. Figure created by the authors using ArcGis 10 (http://www.esri.com/software/arcgis/arcgis-for-desktop).

Even larger changes are found in the case of the NO_3_ radical, the main nocturnal oxidant; Fig. 8 shows a remarkable increase of NO_3_ concentrations of 80% and 32% for the downtown and larger metropolitan areas, respectively. This enhanced oxidation capacity in the nocturnal atmosphere of Madrid has important air quality implications since NO_3_-mediated oxidation of VOCs and organic sulphur species largely influence the budgets of these species and their degradation products^21^. In the particular case of organic sulphur species, H_2_O_2_ is also a powerful oxidizing agent for the aqueous-phase oxidation of S(IV) into S(VI). However, H_2_O_2_ average concentration is found to be very similar in most of the modelling domain, and not to change substantially from 2007 to 2014. Thus, this species does not play a major role in the oxidative capacity changes discussed in the present work. Furthermore, NO_3_ efficiently reacts also with biogenic alkenes^30^ yielding secondary organic aerosols. In most cases, these NO_3_-initiated reactions lead to the formation of organic nitrates, RONO_2_, which represent a considerable fraction of fine particulate matter at the continental scale, and they are found at both urban and rural atmospheres^31^. Thus, the increase in NO_3_ levels in Madrid is very likely to have a profound influence on the production of SOA in the form of organic nitrates. None of the measurements included particulate matter speciation until very recently and the information is too scattered for an analysis of annual mean variation. However, airborne particulate matter concentration was simulated for both inorganic and organic aerosols at the Casa de Campo station, the only one that has a complete and consistent series over the period of interest (see Figure S5 and comments in Supplementary).

**Figure 9:**
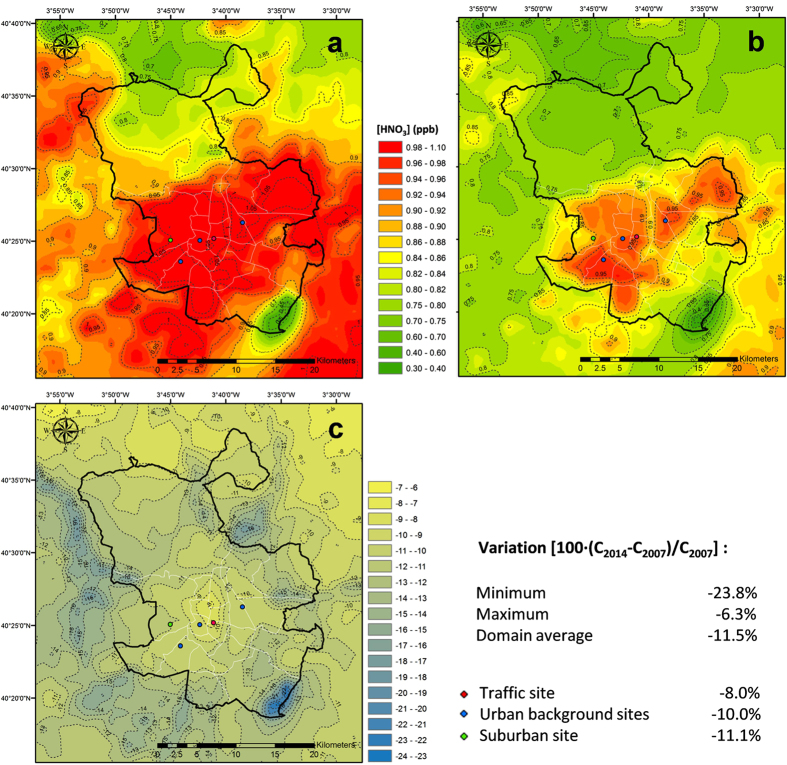
Modelled HNO_3_ annual mean concentration, (**a**) 2007; (**b**) 2014. (**c**) Variation of HNO_3_ levels in 2014 with respect to 2007. Figure created by the authors using ArcGis 10 (http://www.esri.com/software/arcgis/arcgis-for-desktop).

The other important pollutant affected by the reduction in NO_x_ levels is HNO_3_. The increase of OH and NO_3_ radicals may favour the production of HNO_3_ during day and night (via N_2_O_5_), respectively. However, NO_2_ is involved in both production channels and its decline in Madrid’s atmosphere reduces the net formation of HNO_3_. Our results indicate an average reduction between 6–24% in the HNO_3_ concentration over the metropolitan area (Fig. 9). Because of a smaller amount of HNO_3_, the acidification levels of the atmosphere of Madrid and the levels of ammonium nitrate in PM_2.5_ might have been significantly reduced in such a short period. We estimate a reduction in the total oxidized nitrogen deposition of approximately 24% from 2007 to 2014, integrated over the modelling domain.

### Potential influence of meteorological factors

Temperature, precipitation and mixing layer height play an important role in atmospheric chemical reactions and vertical mixing and thus on ambient pollutant concentrations. Average temperature and mixing layer height variations were analysed in order to assess their influence in the observed pollutant trends. A slight positive trend (<0.1 °C year^−1^) of the average annual temperature is observed, although it is not statistically significant (r^2^ of ~0.16). The percentage change in the average annual temperature respect to the 2007 oscillates between 0.04 and 0.09%. The annual averaged mixing layer height also shows a slight positive trend (<15 m year^−1^) but not statistically significant (r^2^ of ~0.28). The annual variation of these parameters is not sufficient to attribute the changes in atmospheric oxidant concentrations to changes in meteorological conditions. However, seasonal and monthly anomalies can contribute to explain some of the observed behaviour. During several winter months (November-December 2007 and 2012, December 2012 and 2014, and January 2012) low wind speeds and strong atmospheric stability episodes lasting 1–2 weeks were registered. These conditions triggered pollution events where NO primary emissions accumulated in the urban area, without an efficient oxidation to NO_2_. These periods, despite being short in time, contribute to a significant increase in monthly means of pollutant concentrations, modifying NO/NO_2_ ratios and in turn perturbing their general trend over these years, as seen in the case of 2011 and 2012 (Fig. S2). Hence, we conclude that the modelled rapid change in the oxidizing capacity in Madrid is not directly related to these mesoscale meteorological factors albeit stability and local winds can play a role in the variability of observed concentrations over the period of interest.

## Discussion

We have shown that the observed increase in O_3_ levels, along with the decrease in NO_x_ emissions over a relatively short period (from 2007 to 2014), may have led to a strong increase in OH and NO_3_ radical concentrations (up to 70% and 90%, respectively), thereby resulting in a rapid and dramatic enhancement in the oxidizing capacity of the urban atmosphere of Madrid. The NO_x_ emission reduction policies and the economic crisis are abating ambient air NO_x_ concentrations, but in turn are introducing substantial changes on atmospheric composition and chemistry that should be taken into account when designing strategies to further improve air quality in urban areas. Lower NO_x_ emissions lead to a strong reduction in HNO_3_ concentrations, and all together to a denoxification of Madrid’s atmosphere and a potential decrease of PM_2.5_ (by decreasing ammonium nitrate formation). We suggest that this has led to a considerable reduction in the nitrogen deposition within and in the surroundings of the metropolitan area that may be relevant for ecosystems.

Our results indicate that the rapid observed trends in O_3_ and NO_x_ would have led to considerable changes in OH and NO_3_ radical budgets, which may have significantly affected the atmospheric chemistry of the metropolitan area of Madrid via (*i*) increased oxidation efficiency of its urban atmosphere, (*ii*) reduction of its atmospheric acidity and (*iii*) enhanced production of secondary pollutants, including SOA. We therefore suggest that this rapidly occurring change in the oxidation efficiency of the Madrid’s atmosphere could also take place in other urban areas in Europe, where current NO_2_ and NO_x_ concentration levels need to be further reduced to meet legal standards. While it is beyond the scope of this paper to estimate the associated radiative effects, NO_x_ reductions are a common aim of air quality plans in urban agglomerations, implying that resulting changes in urban air composition could also have a substantial impact on both regional and global radiative forcing of climate.

Finally, it should be noted that, unlike other pollutants, European NO_2_ standards are particularly restrictive because they follow the World Health Organization guidelines. The results from this case study highlights the need to carefully design and analyze urban air quality plans that usually have the reduction of NO_2_ ambient concentration levels as the primary target. In particular, it is important to fully consider the effect of NO_x_ abatement measures not only on the resulting NO_2_ levels but also on the potential trade-offs with other health-relevant pollutants such O_3_ and aerosols, along with additional environmental impacts such as N deposition to ecosystems.

## Methods

### Measurements

Air quality data were collected from the air quality monitoring network of Madrid. Measurements are based on normalized reference methods described in the European Directive (2008/50/CE)^6^. The equipment works under a rigid maintenance program being periodically tested and calibrated. The air quality monitoring and information system of the Madrid City Council is certified according to ISO 9001, ISO 14001 standards and registered with Eco-Management and Audit Scheme (EMAS). The selection of air quality monitoring stations was based on NO_x_ and O_3_ data availability and consistency of the series over the 2007–2014 period, considering that the monitoring network underwent substantial modifications in 2009 and 2010. The indexes elaborated for this work were computed from over a million of hourly-validated concentration records.

### Analysis of temperature and mixing layer height

Temperature and mixing layer height were obtained from the global meteorological model ECMWF (European Centre for Medium-Range Weather Forecasts). In order to extract meteorological fields for Madrid, a geographical area has been defined between 35° and 45 ° of latitude North and 2° and 5° of longitude West. Daily mixing layer height values are outputed at 12:00 UTC with a spatial resolution of 0.75° (latitude and longitude). In the case of surface temperature, four daily values are used (00:00, 06:00, 12:00 and 18:00 UTC) at a spatial resolution of 0.25°.

### Mesoscale air quality model

The modelling domain covers the entire metropolitan area, i.e. Madrid City and adjacent municipalities that form an urban continuum. This domain consists of 44 × 40 = 1760 1 km^2^-resolution grid cells giving a total area of 1760 km^2^. The AQM is based on the Weather Research and Forecasting (WRF)^26^ (and the CMAQ)^27^,^28^ chemical-transport model. Emissions are processed with the Sparse Matrix Operator Kernel Emissions (SMOKE) modelling system^29^. Further details regarding model options, setup and configuration can be found in a previous work^9^ and references within. We used an emission projection for 2014, since the last year available for the emission inventory was 2012. The reader is referred to Borge *et al*.^9^ for details on emissions scenario computation. CMAQ is a state-of-the-art Eulerian chemistry transport model based on the one-atmosphere paradigm, i.e. solves concentration fields considering the influence of interactions at different dynamic scales and the interactions of the main pollutants and relevant chemical species both primary and secondary. It includes scalable dynamics and thermodynamics (use of fully compressible form of governing equations and a generalized coordinate system) and a modular coding structure with a wide range of representation of scale-dependent processes (e.g. clouds or horizontal diffusivity). The model is applied under a robust nesting approach to consistently describe all the relevant scales involved in urban air quality dynamics. Four nested domains with 48, 16, 4 and 1 km spatial resolution were used to perform the simulations needed in this contribution (see Borge *et al*. for details^9^). The vertical structure of the model is identical to that used for the meteorological model (no layer collapsing techniques were applied) and includes 30 layers covering the whole troposphere (up to 5000 Pa) and high resolution within the planetary boundary layer. The chemical mechanism used is the Carbon Bond 5 mechanism (CB05_AE4_AQ). CB05 is a lumped-structure mechanism that includes 156 gas phase reactions involving 51 species. A full account of species, reactions and corresponding rates can be found in a previous work^32^. Its implementation in CMAQ and a comparison with the previous CB-IV mechanism is described elsewhere^33^. The AERO4 scheme for aerosols is based on ISORROPIA thermodynamic model^34^. Further details regarding chemical mechanism, solvers and other physical options can be found elsewhere^35^.

Emissions are taken from an emission model based on the Sparse Matrix Operator Kernel Emissions (SMOKE) modelling system, described in Borge *et al*.^29^. The model selected to provide the meteorological fields required by the chemical-transport model and the emission processing system is the Weather Research and Forecasting (WRF) modelling system^26^. This non-hydrostatic mesoscale model constitutes a state-of-the-art atmospheric simulation system based on the Fifth-Generation Penn State/NCAR Mesoscale Model (MM5)^36^. A detailed description of the initialization and optimal setup of the WRF model for the Iberian Peninsula can be found in Borge *et al*.^37^. Reference about model validation for different applications can be found in previous works^9^,^35^,^38^,^39^.

### Code availability

SMOKE and CMAQ modelling systems were made available by the US EPA and are supported by the Community Modeling and Analysis System (CMAS) (https://www.cmascenter.org/). The WRF model is a collaborative effort of several research institutions (NCAR, NCEP, FSL and AFWA among others) and is also freely available at the WRF model users’ page (http://www2.mmm.ucar.edu/wrf/users/).

## Additional Information

**How to cite this article**: Saiz-Lopez, A. *et al*. Unexpected increase in the oxidation capacity of the urban atmosphere of Madrid, Spain. *Sci. Rep.* **7**, 45956; doi: 10.1038/srep45956 (2017).

**Publisher's note:** Springer Nature remains neutral with regard to jurisdictional claims in published maps and institutional affiliations.

## Supplementary Material

Supplementary Information
