# Supplementary material for: Unexpected increase in the oxidation capacity of the urban atmosphere of Madrid, Spain
Source: Sci Rep. 2017 Apr 11;7:45956. doi: 10.1038/srep45956 (PMC5387723; doi:10.1038/srep45956)
Supplement: Supplementary Information [file srep45956-s1.pdf]

(Supplementary Information)

Unexpected increase in the oxidation capacity of the urban atmosphere of Madrid, Spain

Authors: A. Saiz-Lopez[1], R. Borge[2], A. Notario[3], C.A. Cuevas[1], J.A. Adame[4] , D. de la Paz[2], X. Querol[5], B. Artiñano[6] and F.J. Gómez-Moreno[6]

[1]Department of Atmospheric Chemistry and Climate, Institute of Physical Chemistry Rocasolano, CSIC, Madrid, Spain.
[2]Laboratory of Environmental Modelling, Department of Chemical and Environmental Engineering, Escuela Técnica Superior de Ingenieros Industriales, Technical University of Madrid (UPM), Madrid, Spain
[3]University of Castilla La Mancha, Physical Chemistry Department, Faculty of Chemical Science and Technologies, Ciudad Real, Spain.
[4]Atmospheric Sounding Station-El Arenosillo, National Institute for Aerospace Technology (INTA), Atmospheric Research and Instrumentation Branch. Mazagón-Huelva, Spain.
[5]Institute of Environmental Assessment and Water Research, CSIC, Barcelona, Spain.
[6]Environmental Department of the Research Center for Energy, Environment and Technology (CIEMAT), Madrid, Spain.

Correspondence to: Alfonso Saiz-Lopez (a.saiz@csic.es)

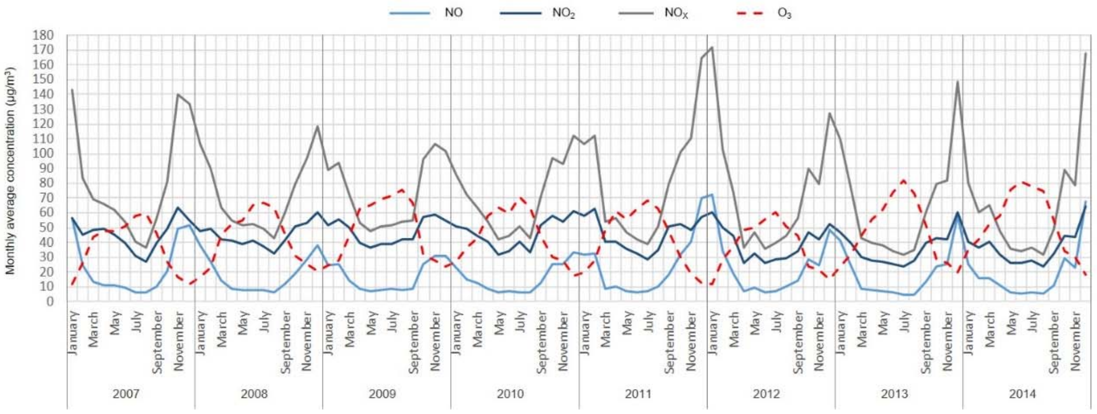

Figure S1. Background NO, NO_2, NOx (as mass of NO_2) and ozone monthly variation.

It can be observed that NOx maxima peaks correspond to greater relative NO increases and these maxima are generally recorded in the months November-January for the years of study period. The occurrence of the highest NOx maxima during these months has been documented to coincide with high stability winter episodes for several years. In order to assess the possible influence in the diminution in ozone annual average levels during 2011-2012, of summer values which clearly down, or values of winter, which also descends in these two years, it can be seen that in the first case in 2011-2012, the summer temperature has been relatively lower (specifically in July which seems to influence in greater extension), and this must influence the lower relative maximum ozone levels observed in summer. Moreover, in those years heavy pollution episodes were registered in winter, where NO levels increased and ozone monthly mean values fell sharply in this season, this fact along the lower production of ozone in summer, leads to a much lower average levels of ozone in that years (please see Figure 4 of the manuscript). Although a clear trend is not observed in the annual mean values of temperature (as above commented), it seems clear the influence of temperature during the month of July in ozone levels.

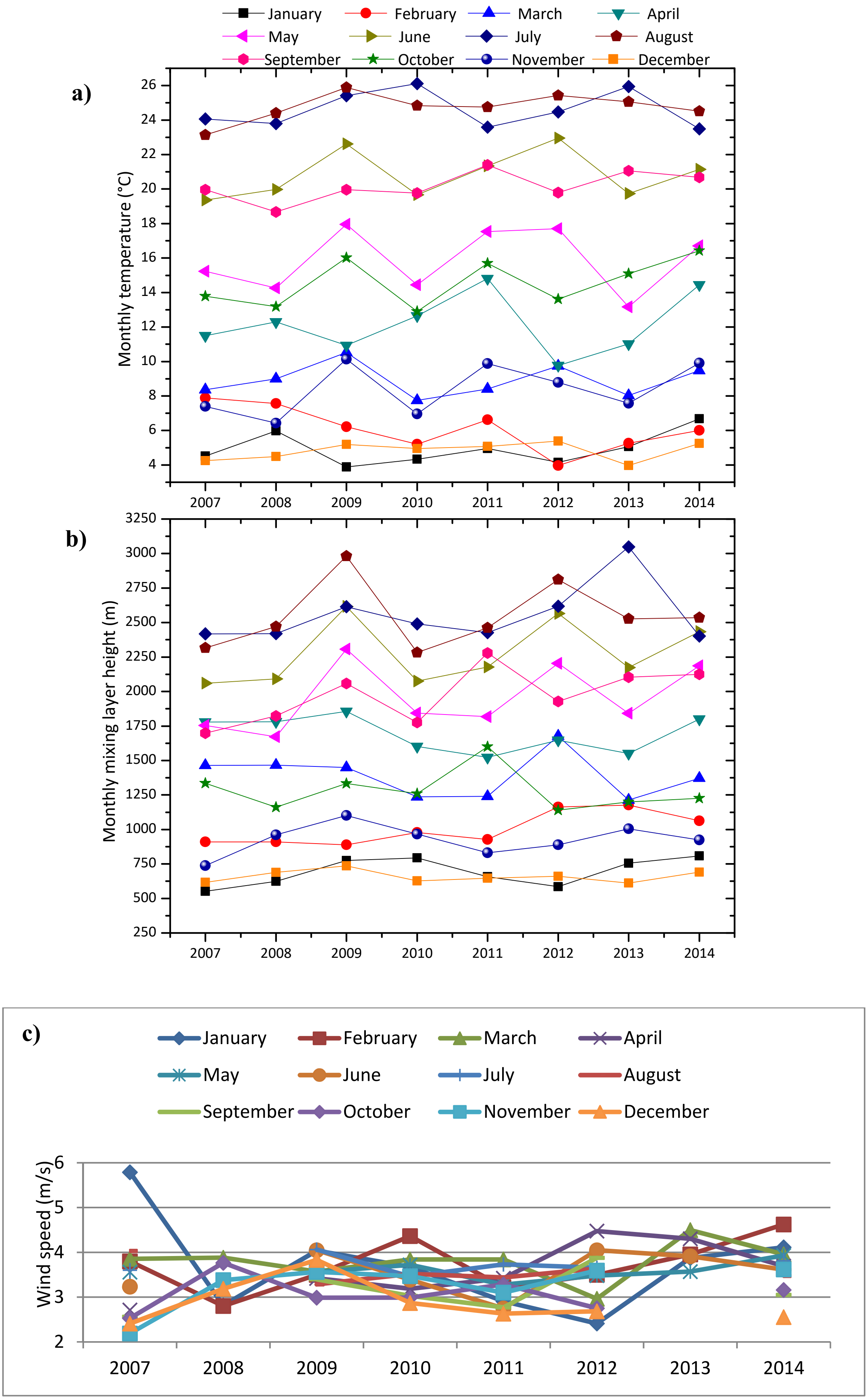

a)
January
February
March
April
May
June
July
August
September
October
November
December
Monthly temperature (°C)
b)
Monthly mixing layer height (m)
2007
2008
2009
2010
2011
2012
2013
2014
c)
Wind speed (m/s)

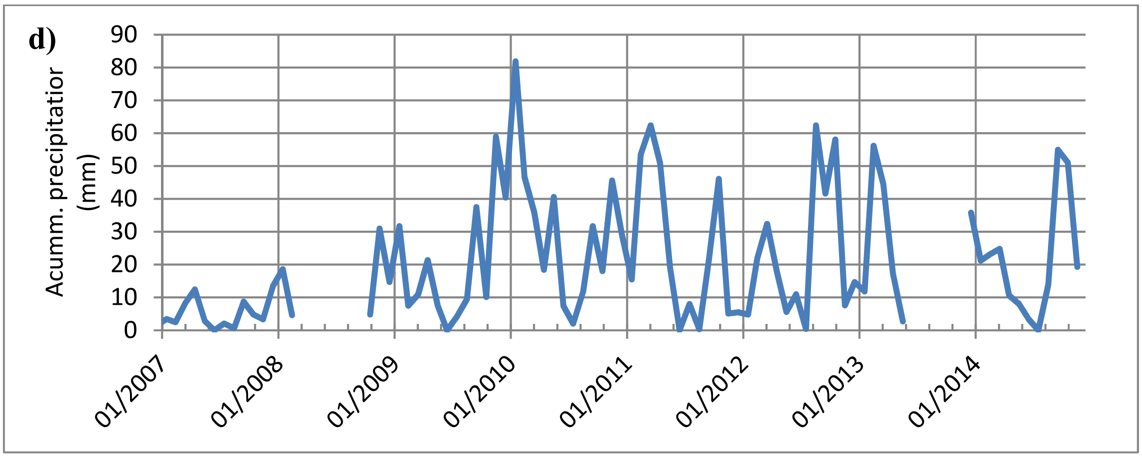

Figure S2. Annual evolution of monthly temperature (a) and mixing layer height (b) (2007-2014) obtained from ECMWF fields for Madrid area, and observed wind speed (c) and accumulated precipitation recorded at CIEMAT* (d). Temperature at 2 m and mixing layer height (ML) has been obtained from the global meteorological model ECMWF (European Centre for Medium-Range Weather Forecasts) with a spatial and temporal resolution of 0.25/0.75 (temperature/mixing layer height) and 6 h respectively. According to the temperature and mixing layer height the year can be divided in four periods. With monthly values of 4-10 ºC and ML lower than 1000 m (November to February), temperature between 10 and 17 ºC and ML of 1000-1500 m (April, May, October); temperature of 18 to 22 ºC and ML of 1500-2500 m (June and September) and monthly temperatures up to 23 ºC and ML up to 2500 m (July and August). They have not found significant differences for these parameters in the studied period.

**The meteorological research station at CIEMAT facilities is an open suburban area NW the city center. This station has four measurement levels and records atmospheric pressure , solar radiation, wind speed and direction, precipitation and temperature at two levels. This station follows the QA/QC protocols imposed by the Nuclear Regulatory Council, which includes two calibrations a year. After a large number of scientific studies over the years this station has been demonstrated to be representative of the atmospheric conditions in the Madrid airshed.*

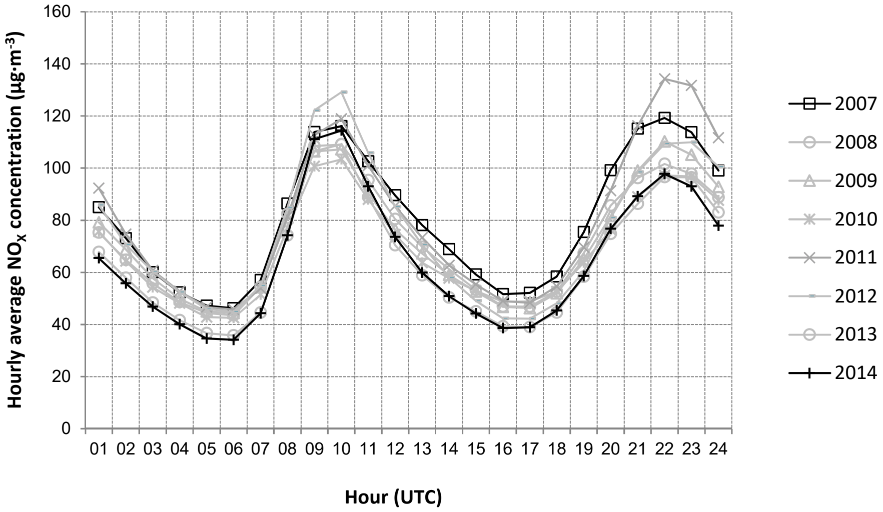

Figure S3. Evolution of the daily average curve of NO_x concentration from 2007 to 2014 in the urban background monitoring stations used in the present work

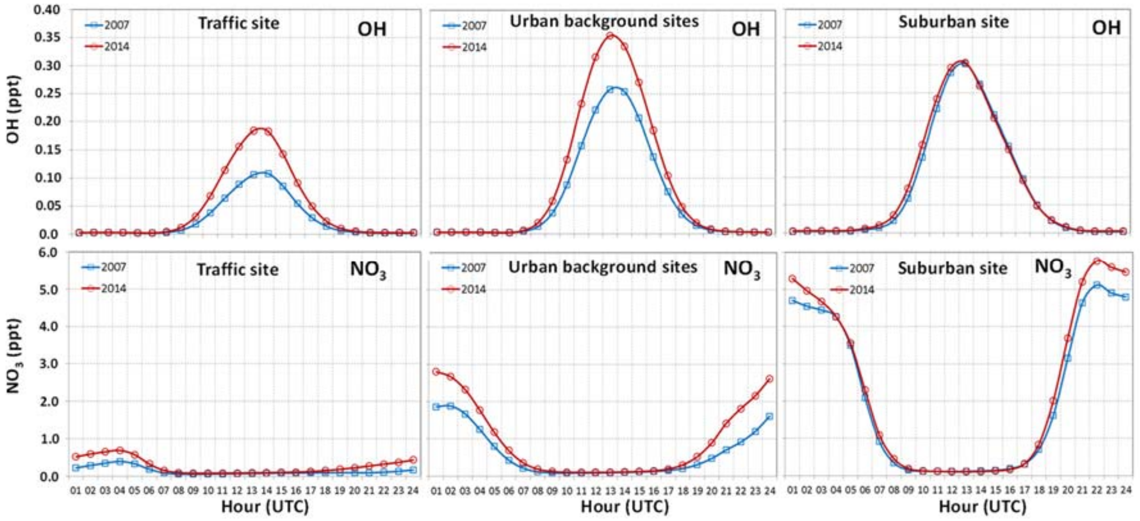

Figure S4. Evolution of the daily average predicted OH and NO_3 concentration levels at the location of representative monitoring stations of Madrid during 2007 and 2014

Daily average concentration patterns requested by the reviewer are shown in Figure S4. It can be seen that the shape of the average concentration curves remains very similar for all the locations and both species in our simulations of 2007 and 2014. OH typically shows virtually null concentration during nighttime and a maximum around midday.

The variation observed for traffic and urban background sites is similar. Maximum OH increments are predicted during the maximum concentration hours. Although absolute increases are slightly larger for urban background locations, relative increment of OH is higher for the reference traffic site where NOx reduction is more pronounced. Moring peak concentrations are found to increase by 78% while this increment for urban background locations is slightly higher than 50%. Differences for the suburban reference site between 2007 and 2014 are minimal with an average increase of 4%.

The typical diurnal variation of the nitrate radical is completely different, with very low values during the daylight hours and maximum concentrations during the night, especially for those locations not directly affected by traffic emissions. Similarly to OH, daily curves are similar for 2007 and 2014. The highest increase in absolute terms is observed also for urban background locations, although the largest relative change is associated to traffic locations, with increments up to 200%. The overall NO_3 increase at the suburban reference site is broadly 10%.

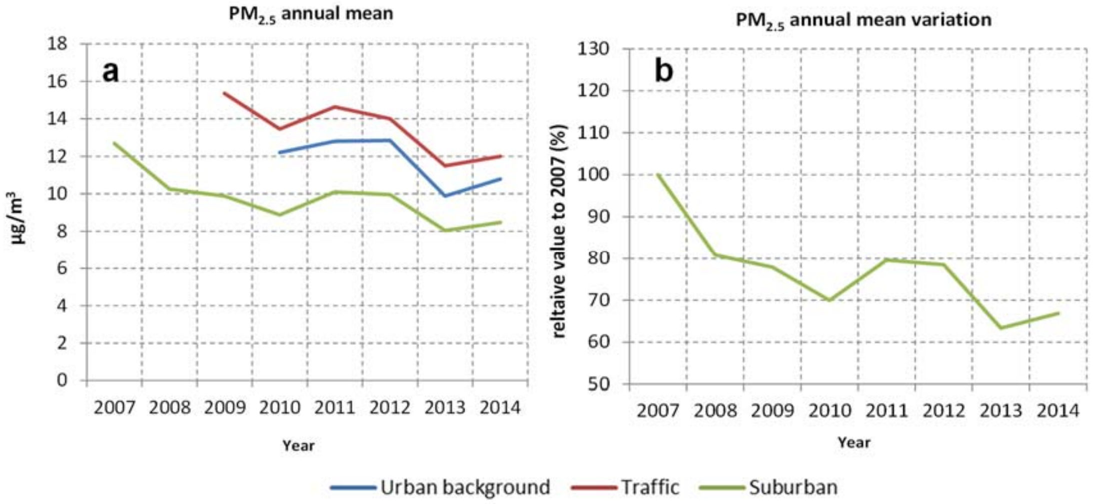

Figure S5. a. Average ambient concentration levels of $PM_{2.5}$ recorded in Madrid during the period 2007-2014. **b**. Percentage variation of $PM_{2.5}$ concentrations respect to levels of 2007 observed at "Casa de Campo".

Actually, the amount of observed $PM_{2.5}$ data is much smaller. Only the reference suburban station selected for this analysis (Casa de Campo) has a complete and consistent series over the period of interest (Figure S5 b). According to the data available, annual mean concentration has dropped by 33% in the 2007-2014 period. The information regarding traffic and urban background locations shown in Figure S5 a, does not correspond to the locations used in this contribution but it is included only to allow a general understanding of $PM_{2.5}$ trends, that seem to follow that observed at the

suburban reference site. None of the measurements included PM speciation until very recently and the information is too scattered for an analysis of annual mean variation.

Airborne particulate matter concentration was simulated as well, both inorganic and organic aerosols (including SOA formation from the oxidation of monoterpenes, long chain alkanes and aromatics). A full description of the aerosol module used in this study is given in Binkowski and Roselle (2003). According to our results, a generalized reduction of total $PM_{2.5}$ is observed (reductions in the range of 5-20% are predicted for most of the modelling domain). Although absolute values are underestimated, the model seems to capture the observed trend, predicting a 10% reduction at the Casa de Campo location. As for $PM_{2.5}$ components, the reduction rate of nitrates ranges from 6 to 10%, i.e. they are substantially reduced but less than primary $PM_{2.5}$ that is reduced by 30% in terms of emissions according to our inventories.

Binkowski, F. S., and S. J. Roselle (2003), Models-3 Community Multiscale Air Quality (CMAQ) model aerosol component: 1. Model description, J. Geophys. Res., 108(D6), 4183, doi:10.1029/2001JD001409.

Impact of VOCs on atmospheric oxidants

It should be noted that VOC and NO_X compete for OH radicals. At low ambient NO_x levels (e.g. NO_x-limited, NO_x-sensitive region), VOCs (including the very reactive oxygenated VOCs[1]) react mainly with OH radicals to produce peroxy radicals that convert NO to NO_2, thereby generating O_3. Therefore, increases in O_3, and associated OH levels, may increase the efficiency of formation of ozone and other secondary photochemical pollutants. As stated before, the increase of O_3 parallel to the NO_2 decrease can be attributed to i) Madrid being a VOCs sensitive region; or ii) urban O_3 concentrations are less depleted due to the lower O_3 titration by substantial NO decreases, as also observed for Paris[2] or suggested for the whole Spain[3]. On the other hand, in Stockholm[2], NO_x reductions leads to decreasing O_3 concentrations; this fact respond to changes in emissions at the regional scale[2]. In the case of Madrid, the VOC (ppb C)/NOx (ppb) concentration ratios during the period of interest are 20.9 and 7.0 for suburban and traffic station, respectively. Nonetheless O_3 concentrations are found to increase as well in this type of location. This may indicate that the chemical response to NOx emissions

abatement is strongly determined by local factors such as and the specific NO/NO_2. In any case the reason of the O_3 increase is out of the scope of this paper and this need to be studied for each specific city.

References

1 Brown, S. S. & Stutz, J. Nighttime radical observations and chemistry. *Chemical Society Reviews* **41**, 6405-6447, doi:10.1039/c2cs35181a (2012).

2 Markakis, K. *et al.* Mid-21st century air quality at the urban scale under the influence of changed climate and emissions – case studies for Paris and Stockholm. *Atmos. Chem. Phys.* **16**, 1877-1894, doi:10.5194/acp-16-1877-2016 (2016).

3 Querol, X. *et al.* 2001–2012 trends on air quality in Spain. *Science of The Total Environment* **490**, 957-969, doi:http://dx.doi.org/10.1016/j.scitotenv.2014.05.074 (2014).
